# Integration of Data from Omic Studies with the Literature-Based Discovery towards Identification of Novel Treatments for Neovascularization in Diabetic Retinopathy

**DOI:** 10.1155/2013/848952

**Published:** 2013-11-24

**Authors:** Ales Maver, Dimitar Hristovski, Thomas C. Rindflesch, Borut Peterlin

**Affiliations:** ^1^Institute of Medical Genetics, Department of Obstetrics and Gynecology, University Medical Centre Ljubljana, 3 Šlajmerjeva Street, 1000 Ljubljana, Slovenia; ^2^Institute of Biostatistics and Medical Informatics, Faculty of Medicine, 1000 Ljubljana, Slovenia; ^3^National Library of Medicine, NIH, Bethesda, MD 20894, USA

## Abstract

Diabetic retinopathy (DR) is a secondary complication of diabetes associated with retinal neovascularization and represents the leading cause of blindness in the adult population in the developed world. Despite research efforts, the nature of pathogenetic processes leading to DR is still unknown, making development of novel effective treatments difficult. Advances in omic technologies now offer unprecedented insight into global molecular alterations in DR, but identification of novel treatments based on massive amounts of data generated in omic studies still represents a considerable challenge. For this reason, we attempted to facilitate discovery of novel treatments for DR by complementing the interpretation of omic results using the vast body of information existing in the published literature with the literature-based discovery (LBD) approaches. To achieve this, we collected data from transcriptomic studies performed on retinal tissue from animal models of DR, performed a meta-analysis of these datasets and identified altered genes and pathways. Using the SemBT LBD framework, we have determined which therapies could regulate perturbed pathways or that could stabilize the gene expression alterations in DR. We show that by using this approach, we not only could reidentify drugs currently in use or in clinical trials, but also could indicate novel treatment directions for ameliorating neovascularization processes in DR.

## 1. Introduction

Diabetic retinopathy (DR) is a secondary complication of diabetes, resulting from microvascular dysfunction and neovascularization in the retinal tissue of diabetic patients [1]. It represents the leading cause of blindness in the adult population in the developed world, and its prevalence progressively increases with duration of diabetes and presence of concomitant features of metabolic syndrome [2]. Despite considerable efforts to define environmental and genetic factors for DR, the exact pathogenetic mechanisms leading to development of this disorder are still poorly understood [3, 4]. For this reason, finding effective treatments represents a significant challenge. Classic treatment with laser photocoagulation remains the modality of choice for prevention of severe visual loss [5]. This approach, however, is only successful in around 50% of cases, while also resulting in restriction of peripheral vision and decrease of color and night vision acuity [5]. Based on recent novel insight into the pathogenesis of DR, novel chemical and biological treatments have emerged, but are either still in the initial stages of clinical utilization or are currently undergoing clinical trials [6]. The main directions of current clinical research into novel medication treatments for DR are focused on using modulators of angiogenesis, nonsteroidal anti-inflammatory drugs, and treatments with steroidal agents [7]. 

Technical advances in the field of molecular biology and molecular genetics, dominated by advances in omic technologies, now offer unprecedented insight into etiology, pathogenetic mechanisms and progression of DR [8–11]. Development of highly parallel technologies now allows investigation of a global profile of alterations in DR on the level of gene sequence, expression, protein alterations, and other molecular levels. In this way, novel contributing mechanisms may be discovered, offering the basis for development of novel treatments acting upon perturbed cellular processes. While omic approaches offer unprecedented insight into etiology, pathogenesis, and homeostatic responses in disease, it is challenging to interpret the large amount of data generated in this manner and to distill the biologically meaningful information, including potential therapeutic targets [12]. For these reasons, novel solutions towards interpreting these results are required to facilitate discovery of mechanisms amenable for therapeutic intervention. 

In order to profitably exploit the latest genomic research techniques, it is essential to use the vast body of information existing in the published literature. Due to the large size of the life sciences literature, sophisticated information management techniques, such as the literature-based discovery (LBD) [13], are needed to complement traditional information retrieval. For example, the LBD paradigm in its essential form can be used to find a new treatment for a disease by first analyzing the literature about the disease to find related pathogenetic processes. The literature is then further searched for substances that address those pathogenetic phenomena and could thus assist in treatment of the disease under investigation. To date, LBD has already been employed on several occasions, aiming to discover new therapeutic agents or to identify new therapeutic targets for existing drugs (drug repurposing)—see Hristovski et al. paper for a review [14]. Several tools allowing LBD-based therapeutics discovery are now available, including two algorithms developed by our group—BITOLA [15–17] and SemBT [18, 19]. The latter tool has been developed to allow incorporation of data from omic technologies into the primary LBD search mechanism and allows identification of therapeutics based on empirical data originating from various omic approaches, including results from transcriptomic, proteomic, and other studies. 

We therefore hypothesized that discovery of novel therapeutics for neovascularization processes in DR based on data generated by omic technologies could be facilitated by incorporation of information from LBD. To demonstrate this, we have collected the data on transcriptomic alterations occurring in animal models of DR and attempted to find new therapeutic modalities based on this source of omic data using the LBD approach.

## 2. Methods and Materials

To demonstrate the feasibility of information provided by global molecular profiling approaches in searching for novel potential therapeutic targets for DR, we have selected available data from previously performed genome-wide expression (transcriptome) profiling studies in DR and performed a meta-analysis of these datasets. Afterwards, a search for therapeutics with potential for use in DR was performed on this data with support from the LBD approach implemented in the SemBT tool. 

### 2.1. Meta-Analysis of Transcriptomic Alterations in Retinas of Animal Models with Diabetic Retinopathy

Initially, a search and selection of amenable studies were performed based on data deposited in the Gene Expression Omnibus (GEO) and ArrayExpress (AE) databases. Studies found were in all cases performed on retinal tissue from animal models of DR, where diabetes was artificially induced by streptozocin (Table 1).

Subsequently, studies were selected for meta-analysis based on sufficient number of samples and compatibility of study design. Based on these criteria, two studies (GSE19122 and GSE12610) reporting transcriptome alterations in mouse models of DR were incorporated in the meta-analysis in order to determine a set of most consistently differentially expressed genes in retinal samples of animal models of DR. 

All the following steps in this section were performed in the R statistical environment version 2.7.1 (http://cran.r-project.org/), in the Bioconductor environment (available at http://bioconductor.org/, [20]). Raw data from microarray experiments were obtained from the GEO repository (http://www.ncbi.nlm.nih.gov/geo/, [21]) and were examined using the arrayQualityMetrics package, followed by normalization and nonspecific filtering with affyPLM and genefilter packages, where necessary. Ultimately, 12,177 genes with expression values measured for 19 samples (10 mice with DR and 9 controls) met our filtering criteria and were included in the meta-analysis step. 

Differential expression of genes across all three studies was calculated using meta-analysis algorithms implemented in the RankProd package [22]. RankProd uses a nonparametric statistical measure to detect genes constantly highly ranked across different microarray datasets and is therefore a feasible meta-analysis tool, enabling fusion of omic data from different studies and allowing for inclusion of data from different laboratories and performed on differing platforms. Significance values and false discovery rate (FDR) values were calculated by performing 1000 permutations of the source dataset. Mouse gene Entrez identifiers were then converted to their human counterparts using homology information for mouse genes collected in the *hom.Mm.inp* Bioconductor annotation library. Human orthologs of mouse genes with highest differential expression in mouse models of DR were therefore included as targets for novel therapeutic discovery by the SemBT algorithm.

We also performed gene set enrichment analysis of genes scoring highest in meta-analysis of two datasets against a background of all human genome genes; for this reason Gene Ontology functional gene annotations [23] were utilized, and the DAVID tool (http://david.abcc.ncifcrf.gov/, [24]) was used for estimating the enriched functional categories, where overrepresentation was called after the significance scores were below 0.05 after adjustment for multiple testing according to Benjamini-Hochberg correction [25].

### 2.2. Searching for Novel Candidate Therapeutics Using the Literature-Based Discovery

**Figure 1:**
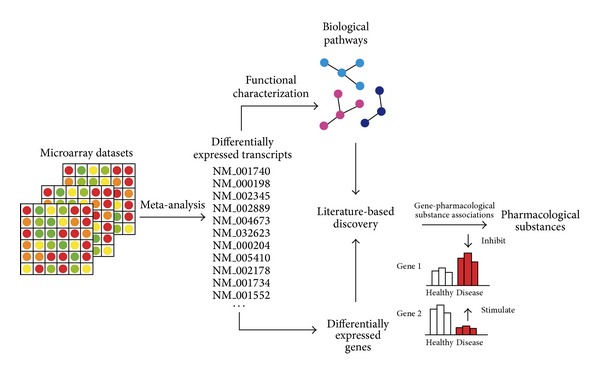
Workflow of approaches employed to identify novel therapeutics for DR based on transcriptional profiling information.

We considered two paths towards finding novel therapeutics based on whole transcriptome profiling information (Figure 1). Firstly, we searched for pharmacological substances modulating pathways altered in DR models, and secondly, we directly searched for substances that have a stabilizing effect on the largest set of genes in models of DR. The complete process of analyses workflow in the present study is presented schematically in Figure 1. 

To search for novel therapeutics based on transcriptomic data, we utilized the SemBT tool developed by Hristovski et al. [18, 19], available in [26], allowing us to integrate the results of microarray gene expression experiments with semantic relations extracted from the literature with the SemRep [27] application. SemRep is symbolic rule-based natural language processing system that extracts semantic predications from MEDLINE citations in several domains, including clinical medicine [27], molecular genetics [28], and pharmacogenomics [29]. In SemBT, we used the data generated by microarray expression profiling to obtain information on which genes are upregulated and which are downregulated in DR, compared to control subjects. The semantic relations provided information on the interactions of these differentially expressed genes with other biomedical concepts.

**Figure 2:**
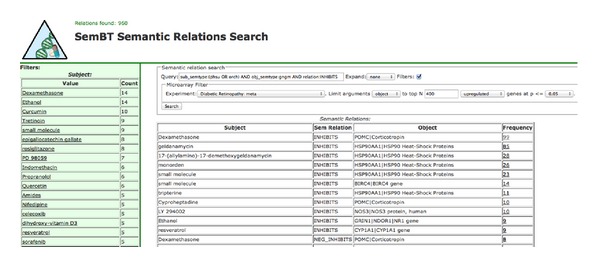
Finding agents that inhibit a subset of genes up-regulated in diabetic retinopathy.

For integration of omic data and LBD, we have developed *discovery patterns, *which are query combinations whose results represent a novel hypothesis—not evident in the literature or in the microarray results alone. For example, to investigate genes that are upregulated in the microarray, we searched for concepts (genes, drugs, etc.) that are reported in the literature as inhibiting the upregulated genes. We call this discovery pattern “*inhibit the upregulated*.” Similarly, we investigated downregulated genes with the “*stimulate the downregulated*” pattern, in which case we searched for biomedical concepts known to stimulate the downregulated genes. Using these discovery patterns, we could combine information from the microarray data about up- or downregulated genes in patients having DR with information from the literature about biomedical concepts that can be used to regulate those genes. An example of a search using these discovery patterns is shown in Figure 2, which implements the “inhibit the upregulated” pattern in SemBT for DR. In the *Query* field, in a suitable syntax, we limited the search to pharmacologic substances (drugs) or organic chemicals that can inhibit target genes or their products. Then, in the *Microarray Filter* group of fields, we require that those same genes that are inhibited are those that are upregulated in DR. 

After the search was executed, two groups of results were generated by SemBT. The group *Semantic Relations* reflects particular drugs inhibiting genes that are upregulated; sorted in descending order by the number of times (*Frequency* field) the semantic relations were mentioned in the biomedical literature. For each association, SemBT provided a hyperlink, which was used to show the list of sentences from which the relations were extracted. Additionally, SemBT provided the PubMed ID (PMID for each sentence; this was used to show the PubMed citation in which the sentence appears). Examples of this aspect of SemBT are shown in Table 2. The second group of results reflects the number of distinct genes, a particular drug inhibits or stimulates, depending on the discovery pattern used for search. In Figure 2, these results are shown in the left column (obtained by using *Filters* option in the SemBT interface). 

## 3. Results

We performed a meta-analysis of available transcriptomic data in animal models of DR. Firstly, the search was carried out for therapeutics affecting perturbed pathways, identified by transcriptome profiling, and subsequently, for therapies directly modulating genes with altered expression in DR. 

### 3.1. Results of Meta-Analysis of Genome-Wide Expression Profiling in Diabetic Retinopathy

Meta-analysis of included studies has revealed 385 genes upregulated and 539 probesets downregulated in DR animal models with false positive rate values lower than 0.05. Significantly enriched functional categories of genes, annotated with terms from the Biological Process branch of the Gene Ontology, are presented in Table 3. The result of functional profiling for top genes included pathways with a well-established role in DR, such as regulation of cell proliferation and angiogenesis.

### 3.2. Identification of Therapeutics Affecting Pathways Altered in Diabetic Retinopathy

To demonstrate the feasibility of a pathway-based approach to find therapeutic modalities suitable for treatment, we initially searched for therapeutic agents modulating the vasculogenesis pathway. The results of this search included therapeutic substances with a previously described therapeutic effect in DR, including a group of therapeutics belonging to the class of angiogenesis inhibitors (including bevacizumab), which are currently either in initial phases of clinical applications or being investigated in clinical trials (Table 4). Interestingly, the potential of anti-inflammatory treatments has already been noted in this stage, with the high ranking of nonsteroidal anti-inflammatory agents and dexamethasone among the top candidate therapeutic agents. 

Following this search, we performed a combined search for therapeutic substances modulating main functional pathways discovered by transcriptomic profiling in the retina. We have searched for substances concurrently modulating several pathways found altered in DR. The results of such a search are presented in Table 5. Here, in addition to angiogenesis modulators the algorithm also identified therapeutic agents modulating angiogenesis and cell proliferation processes concurrently. 

### 3.3. Identification of Therapeutics with a Stabilizing Effect on Genes with Altered Expression in Diabetic Retinopathy

We searched for therapeutic substances conferring a stabilizing effect on gene expression, causing downregulation of genes upregulated in DR and upregulation of genes downregulated in DR. The therapeutic substances stabilizing expression of the largest number of genes towards baseline are presented in Table 6. 

Dexamethasone in addition to nonsteroidal anti-inflammatory agents was found to stabilize the largest number of genes differentially expressed in DR, concurrently conferring a stabilization effect on both downregulated and upregulated genes. Additionally, antioxidants were found to stabilize a large number of genes differentially expressed in DR. 

Both approaches to novel therapeutics identification demonstrated the possible utility of a set of known drugs or substances, including dexamethasone, curcumin, resveratrol, and tretinoin, which affect pathways perturbed in DR while at the same time stabilizing the specific gene expression alterations occurring in DR. 

## 4. Discussion

We have shown the feasibility of LBD to support finding novel therapeutic targets, based on information generated by global transcriptional profiling in a model of DR. We have shown that hypothesis-free approaches may detect both previously known and novel pathogenetic mechanisms occurring in the disease model of DR. We have also demonstrated that literature-based discovery could predict treatments that are either already utilized for treatment of DR or may present novel therapeutic opportunities. 

In the present study, we have advanced the approach that we previously utilized for discovery of novel therapeutics, by allowing for identification of substances targeting pathways altered in DR and substances stabilizing the expression of genes altered in DR. Although several studies have utilized omic technologies to investigate global alterations in DR, so far most comprehensive studies have been performed on animal models, specifically on the level of transcriptomic alterations in DR, which directed us to use this information in searching for novel treatments for DR. To show the utility of LBD in discovering novel treatments based on data from transcriptome profiling approaches, we initially collected data from studies performed on animal models of DR and performed a meta-analysis of these datasets. Among the top genes uncovered by the meta-analysis, genes related to functions known to be dysregulated in DR were detected, including cell proliferation processes and vasculature development. Considering that the process of neovascularization is a hallmark of advanced DR and includes cellular proliferation and development of new blood vessels in the retina, we believe that the transcriptome could be regarded as a useful surrogate for biologic processes occurring in human disease. 

The search we used for therapeutic agents in DR also has some pitfalls. Firstly, the source studies we utilized in the search were performed on animal models of DR after artificial induction of diabetes by streptozocin, which may not represent a faithful surrogate for pathogenetic processes occurring in human cases of DR. Despite this, we expect that more comprehensive omic studies in human DR will be conducted in the near future, allowing the possibility for even more effective therapeutics search based on such data. Secondly, as the discovery of associations between therapeutic agents and differentially expressed genes is performed by computational language processing, this may result in the generation of spurious associations and false positive predictions of possible therapeutic substances. This effect may, however, be ameliorated by inspection of sentences supporting the associations prior to definitely assuming the therapeutic potential of the substance. Thirdly, we have noted that substances with more publications in the literature tend to occur more commonly in the list of top potential treatment substances. To ameliorate this issue, it would be possible to prioritize potential substances based on a representation scoring approach, normalizing the publication bias. 

Although LBD has already been used to generate hypotheses regarding novel therapeutic approaches (see [14] for a review), most of the approaches are based purely on the literature, and in only one case has LBD been integrated with transcriptomic experiment results [18]. In the approach described in this paper, we additionally integrate into LBD the results of gene enrichment analysis based on Gene Ontology functional categories, which to our knowledge, has not been done before. Furthermore, LBD has not been applied to DR before.

In conclusion, the work presented incorporates a novel strategy towards identification of treatments for human disease based on integration of data from omic technologies with LBD and provides novel therapeutic directions for treating neovascularization processes occurring in DR. 

## Figures and Tables

**Table 1:** List of studies reporting alterations in global expression profile in DR.

GEO accession	Species	Description	Array platform	Sample number	Ref.
GSE19122	Mouse	Streptozotocin (STZ) induced	Illumina Ref8	14	[11]
GSE12610	Mouse	STZ induced	Affymetrix mouse genome 430 2.0 array	5	NA
GSE20886	Rat	STZ induced	Illumina ratRef-12	9	[10]
GSE28831	Rat	STZ induced	Agilent-014879 whole rat genome microarray	6	[30]

NA: no journal article could be associated with GEO identifier.

**Table 2:** Some of the sentences from which the semantic relation Resveratrol-inhibits-CYP1A1 was extracted.

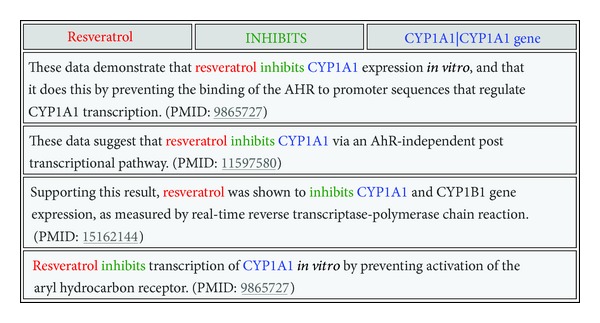

**Table 3:** The highest ranked GeneOntology terms, annotating top genes resulting from the meta-analysis of microarray studies performed in rat models of diabetic retinopathy.

GeneOntology term	*P* value*
Regulation of cell proliferation	2.5*E* − 4
Intracellular signaling cascade	7.4*E* − 3
Negativee regulation of macromolecule biosynthetic process	2.9*E* − 2
Response to wounding	3.0*E* − 2
Negative regulation of multicellular organismal process	3.9*E* − 2
Vasculature development	4.3*E* − 2

*Significance values for enrichment are adjusted for multiple testing according to the Benjamini-Hochberg method.

**Table 4:** Pharmacological substances affecting the angiogenesis pathway found dysregulated by global gene expression profiling in diabetic retinopathy models. Presented are pharmacological substances having at least 20 or more associations with the angiogenesis pathway in the literature.

Subject	Semantic relation	Object	Frequency
Endostatins	Inhibits	Angiogenesis	69
Thalidomide	Inhibits	Angiogenesis	37
Angiostatins	Inhibits	Angiogenesis	34
Curcumin	Inhibits	Angiogenesis	30
Bevacizumab	Inhibits	Angiogenesis	29
TNP 470	Inhibits	Angiogenesis	23
Sphingosine 1-phosphate	Affects	Angiogenesis	22
Small molecule	Inhibits	Angiogenesis	22
Epigallocatechin gallate	Inhibits	Angiogenesis	21
Anti-inflammatory Agents, Nonsteroidal	Inhibits	Angiogenesis	16
Resveratrol	Inhibits	Angiogenesis	15

**Table 5:** Pharmacological substances affecting the main biological pathways found dysregulated in diabetic retinopathy models by transcriptional profiling in disease.

Substance	Semantic relation	Process	Frequency
Curcumin	Disrupts	Cell proliferation	69
Endostatins	Inhibits	Angiogenesis	69
Tretinoin	Disrupts	Cell proliferation	65
Resveratrol	Disrupts	Cell proliferation	56
Dexamethasone	Disrupts	Cell proliferation	52
Tretinoin	Affects	Cell proliferation	50
Caffeine	Disrupts	Wound healing	45
Epigallocatechin gallate	Disrupts	Cell proliferation	43
Polyamines	Affects	Cell proliferation	40
Thalidomide	Inhibits	Angiogenesis	37

**Table 6:** Pharmacological substances with a potential stabilizing effect on genes differentially expressed in animal models of DR. Substances in bold are those occurring in both lists, concurrently stabilizing both upregulated and downregulated genes.

Therapeutic substance	Number of downregulated genes in RD that are stimulated by the substance
**Dexamethasone **	19
**Tretinoin **	18
**Ethanol **	12
Phenylephrine	7
**Curcumin **	6
Hydroxymethylglutaryl-CoA reductase Inhibitors	6
Isoproterenol	6
Resveratrol	6

Therapeutic substance	Number of upregulated genes in RD that are inhibited by the substance

**Dexamethasone **	14
**Ethanol **	14
**Curcumin **	10
**Tretinoin **	9
Small molecule	9
Epigallocatechin gallate	8
Rosiglitazone	8
PD 98059	7
Indomethacin	6
Propranolol	6
Quercetin	6
